# Gender differences in attitudes towards psychological help-seeking among chinese medical students: a comparative analysis

**DOI:** 10.1186/s12889-024-18826-x

**Published:** 2024-05-15

**Authors:** Lei Qiu, Hengyi Xu, Yingqi Li, Yonghui Zhao, Qin Yang

**Affiliations:** 1 School of Public Health, Hainan Medical University, Hainan, People’s Republic of China; 2 Institute of Social Development and Health Management, Hubei Province Key Laboratory of Occupational Hazard Identification and Control, School of Public Health, Wuhan University of Science and Technology, Wuhan, People’s Republic of China; 3 School of Management, Hainan Medical University, Hainan, People’s Republic of China; 4 Xiangyang Central Hospital, Affiliated Hospital of Hubei University of Arts and Science, Xiangyang, People’s Republic of China

**Keywords:** Attitude, Mental health, Help-seeking behavior, Chinese medical students

## Abstract

**Background:**

Medical students are known to be at a greater risk of psychological disorders compared to the general population. However, their rate of help-seeking behavior is low. The purpose of this study was to explore the influencing factors of attitudes towards psychological help-seeking among Chinese medical students and to examine its gender differences.

**Methods:**

A total of 3,453 medical students from three medical colleges in Hainan Province, China, completed anonymous questionnaires that included socio-demographic attributes, the Family APGAR Index, the General Health Questionnaire (GHQ-20), and the Attitudes Towards Seeking Professional Psychological Help Short Form (ATSPPH-SF). Associations between predictor variables and attitudes towards help-seeking were explored using multivariate linear regression, and regression models with interaction terms were employed to test gender difference.

**Results:**

The mean score on ATSPPH-SF Scale was 15.04 ± 3.45, with males scoring significantly lower than females (14.34 vs. 15.64, *P* < 0.0001). For both male and female groups, psych knowledge, mental health status, family function and help-seeking utility perception significantly influenced attitudes toward psychological help-seeking. Furthermore, having more than once psycho-help experiences was positively correlated with women’s attitudes. Significant interactions were found between gender and mental health status.

**Conclusion:**

Attitude towards seeking psychological help was relatively negative among Chinese medical students. The implementation of interventions should take into account the at-risk population, especially the males and individuals with poor mental health.

## Introduction

The significance of mental health for maintaining overall wellbeing cannot be overstated, particularly in high-pressure educational contexts such as medical schools [1, 2]. Due to the rigorous academic demands and emotionally challenging clinical encounters, e.g., exposure to patients’ deaths, medical students were at increased risk of suffering from psychological distress [3–7]. A meta-analysis of 167 studies indicated that 27.2% of medical students had depression and 11.1% had suicidal ideation [8]. Another meta-analysis estimated that the prevalence of anxiety among medical students was 33.8% [9]. Seeking professional psychological help is key to mitigating these psychological burdens. Professional psychological help refers to the provision of therapeutic interventions and support by trained and licensed mental health professionals. This assistance encompasses various forms of psychotherapy, counseling, and psychological assessment aimed at addressing individuals’ emotional, behavioral, and mental health concerns. Professionals offering such help may include psychologists, psychiatrists, clinical social workers, counselors, and therapists.

However, only a minority of medical students would seek psychological help. Prior research revealed that less than 16% medical students with depression sought treatment [8]. Reasons for not seeking help include limited time, stigmatization, concerns about confidentiality and treatment costs [10]. Especially, the attitudinal barrier is one of the main obstacles to receiving treatment [11]. The delayed psychotherapy caused by negative attitudes not only impedes their recovery from psychological disorders, but also induces some physical diseases, ultimately reducing the quality of life. For instance, depression has been identified as a risk factor for many chronic diseases, e.g., diabetes mellitus, heart disease and stroke [12]. Within the context of medical students, this issue was even more crucial. Help-seeking attitudes among this group could not only affect their personal wellbeing but also their academic achievement [13] and future medical careers, such as increasing the probability of making medical errors [14–16].

Evidence suggests that attitudes towards seeking psychological help can be influenced by various factors, such as individual, sociological and cultural factors. Individual factors encompass age [17], gender [18, 19], educational level [20], work status [18], and public/social stigma as well as self-stigma [18, 21, 22]. Furthermore, normative alexithymia and fear of intimacy [20] have been found to be associated with a negative attitude towards help-seeking. Individuals who have previously received psychological treatment tend to have a more positive attitude towards seeking help [23]. Additionally, traditional masculine ideology [20] has been identified as a significant factor influencing attitudes towards seeking help. Among these factors, gender differences have garnered significant attention from scholars due to their pivotal role.

This gender difference can be explained by traditional masculine gender norms and stereotypes, such as self-reliant, being strong and emotional control [24, 25]. Men were more likely to perceive psychological treatment as a sign of weakness [26]. A cross-sectional study from United Kingdom confirmed that a higher level of traditional masculine ideology was significantly associated with more negative attitudes towards psychological help seeking [20]. On the contrary, from the perspective of social psychology, women are more willing to disclose themselves than men [27]. Another factor contributing to gender differences in attitudes towards seeking psychological help may be that the incidence of psychological disorders in women is significantly higher than in men [28, 29], and the symptoms are generally more severe than in men. For example, compared to men with depression, women with depression typically experience longer periods or repeated episodes of low mood and experience greater weight gain [30–32]. More severe clinical symptoms force women to confront psychological distress and try to find solutions.

Although attitudes towards professional psychological help-seeking have attracted considerable attention from scholars, research on this topic in China remains insufficient. There is little research investigating the attitudes towards professional psychological help-seeking among Chinese medical students and its influencing factors. Furthermore, the gender differences in the attitudes among medical students in a Chinese context are largely unknown. Therefore, our study has the following three purposes. Firstly, investigate the attitudes towards psychological help-seeking among Chinese medical students. Secondly, assess the gender difference in seeking help attitudes. Thirdly, explore the influencing factors of attitudes in male and female medical student groups respectively, as well as test its gender difference in the context of China. By addressing this difference, we hope to contribute to a more nuanced understanding of gender-related barriers to psychological help among medical students and provide scientific evidence for intervention measures and policy making.

## Method

### Study design and setting

We conducted a cross-sectional study from January 1, 2021, to May 31, 2021, within Hainan Province, China, which encompasses the Hainan Island, Paracel Islands, the Spratly Islands, and the Zhongsha Islands.

### Sample selection

After identifying the three medical colleges and their respective majors, we detailed the composition of classes and students as follows: Hainan Medical University, with its 11 majors, has classes ranging from 1 to 4 per academic year, culminating in a total of 110 classes with 5,280 students. Similarly, Hainan Health Management College and Hainan Health Vocational College have a structured distribution across their 8 and 7 majors, respectively, with 80 and 69 total classes, and 3,841 and 2,898 students.

Participants were selected using a stratified cluster sampling design. Firstly, to ensure a comprehensive analysis across the spectrum of medical education, we strategically selected five medical majors from each of the three institutions. This decision was informed by the objective to capture a diverse range of student experiences and educational backgrounds. Due to some overlap in majors among the three medical schools, a total of 11 distinct majors were selected, including preventive medicine, medical laboratory technology, clinical medicine, nursing, anesthesia, imaging, pharmaceutical management, etc. Secondly, the selected majors were divided into one to four levels by grade, and one class was randomly selected from each level. Thirdly, all students from the selected class will be recruited as research participants. To maintain the integrity of our random sampling method, a computer-generated random number sequence was used for the selection of both majors and classes. This process ensured an unbiased and equitable chance of selection for every major and class within the specified academic levels across the three institutions.

## Data collection

The data collection took place in classrooms, with the selected classes assembled in a designated setting. Trained investigators distributed the questionnaires to students and subsequently collected them upon completion. To ensure data integrity, two members of the research team cross-checked the entries for accuracy, after which the data were entered into the EpiData 4.6.0.0 software.

### Sample size and response rate

The sample size for our study was estimated using formula (1):

1 $$N=\frac{{t}^{2}p(1-p)}{{e}^{2}}$$

*N*: the sample size; *t*: the critical value for the significance test at a confidence level of 99%, i.e. *t* = 2.56; *p*: assuming a population proportion of 4%, i.e. *p* = 4%; *e*: the allowable error, with a sampling error range of no more than 1%, i.e. *e* = 1%. The calculated sample size was 2,517 participants. Considering a 10% questionnaire invalidation rate, the minimum required sample size should be 2,797.

In the aforementioned process, a total of 3,596 medical students from the three institutions were invited to participate in the survey, with 126 refused. Ultimately, we collected 3,470 questionnaires, with a commendable response rate of 96.49%. Out of the collected questionnaires, 17 were found to have inconsistencies or significant missing data and were thus excluded, resulting in 3,453 valid questionnaires.

### Measuring instruments

Prior to the main study, a pilot survey was administered to 60 medical students from Hainan Medical University. The pilot study primarily aimed to evaluate the comprehensibility of questionnaire items by students, identify and address any ambiguities, gauge student receptiveness towards the questionnaire, and evaluate completion time. Following the pilot survey, revisions and enhancements were made to the questionnaire based on feedback and suggestions from the surveyed individuals, resulting in the final version of the survey instrument.

The structured questionnaire, distributed for self-administration, was segmented into four primary divisions: (1) Socio-demographic attributes, (2) Family APGAR Index, (3) General Health Questionnaire (GHQ-20), and (4) Attitudes Towards Seeking Professional Psychological Help Short Form (ATSPPH-SF). Within the demographic data, parameters such as age, gender, sibling status, major, prior psycho-help experiences, Help-seeking utility perception, and self-rated psych knowledge were collected. Prior psycho-help experience is assessed with the question, “Have you ever received psychological counseling or professional psychological assistance during your college years?”, and the responses are categorized as None, Once, or More than once). Help-Seeking Utility Perception is assessed with the question, “Do you believe psychological counseling is helpful?”, and the responses are categorized as Very useful, Somewhat useful, Neutral, Somewhat, Not useful, or Not useful at all. Self-Rated Psych Knowledge is assessed with the question, “Please rate your understanding of psychology knowledge on a scale from 1 to 10, where 1 indicates no understanding at all, and 10 indicates a very high level of understanding.”, and the responses are divided into low level (1–7) and high level (8–10).

The Family APGAR Index was developed by Smilkstein in 1978 [33] and used to measure family function. This index comprises five items encapsulating the facets of adaptation, partnership, growth, affection, and resolve. Items are evaluated utilizing a 3-point Likert Scale: 0 corresponding to “almost never”, 1 to “sometimes”, and 2 to “often like this”. The total score is obtained by adding up the scores of 5 items, with a range of 0–10. The higher the score, the better the family function. And a score of 7–10 indicates good family function, 4–6 indicate moderately impaired family dysfunction; and 0–3 indicate severe family dysfunction. In the Chinese context, the Family APGAR Index boasts extensive application and has manifested commendable reliability and validity [34]. Notably, in the current investigation, the index recorded a Cronbach’s alpha of 0.88.

The GHQ-20 was initially formulated by Goldberg [35] and subsequently revised by Dr. Li Hong and colleagues from Tsinghua University [36]. This instrument is comprised of 20 items distributed across three subscales: GHQ Self-Affirmation (9 items), GHQ Depression (6 items), and GHQ Anxiety (5 items). The scoring for the GHQ-20 is binary, where participants determine the applicability of each item to themselves, with “Yes” corresponding to 1 point and “No” equating to 0 points. Elevated scores on the Depression and Anxiety scales signify heightened levels of respective symptoms, whereas an increased score on the Self-Affirmation scale denotes a robust degree of self-affirmation. The aggregate score is derived by summing the scores from all three subscales, with the caveat that the Self-Affirmation scale is inversely scored. A greater cumulative score is indicative of elevated psychological distress. Among Chinese students, the GHQ-20 has shown commendable reliability and validity, with the internal consistency ranged from 0.60 to 0.82, and the correlation validity of its subscales ranged from 0.32 to 0.67 for the scale and subscales [36]. The Cronbach’s alpha of GHQ-20 is 0.83 in this research.

The ATSPPH-SF is a 10-item scale, which was a shorter version developed from Fischer and Turner’s 29 item ATSPPH scale (1970) [37]. Each item of the scale is scored on a 4-point Likert Scale ranging from 0 = “Strongly disagree” to 3 = “Strongly agree”. Participants selected the point he/she agreed with the statement (e.g. Personal and emotional troubles, like many things, tend to work out by themselves) to reflect his/her own attitude towards seeking professional psychological assistance. Items 2, 4, 8, 9 and 10 are scored in reverse and the total score is obtained by adding up the scores of all items, with a range of 0 to 30. A higher total score indicating a more positive attitudes towards seeking help. In the context of China, the ATSPPH-SF has been proven to be valid and reliable, with a Cronbach’s alpha range from 0.66 to 0.71 and the test-retest reliability ranged from 0.78 to 0.90 for the scale and subscales [38]. In our study, the Cronbach’s alpha of the ATSPPH-SF was 0.69.

### Statistical analysis

The Statistical Analysis System (SAS) 9.4 for Windows (SAS Institute Inc., Cary, NC, USA) was used for statistical analysis. Firstly, descriptive analyses were carried out for all independent variables and reported means, standard deviation, frequency and percentages. Secondly, for positively worded items of ATSPPH-SF scale, the options of “Agree” and “Strongly agree” were combined into one option, while for negatively worded items, the options of “Disagree” and “Strongly disagree” were combined into one option. Frequencies, proportion and it’s 95% CI were calculated for each merge option. Disparities between genders for each item were probed using Pearson’s Chi-square test. Mean values and standard deviations of cumulative scores were documented. Furthermore, Pearson correlation analyses were employed to discern gender-based variations in cumulative scores. Three distinct models were crafted to evaluate determinants influencing attitudes towards psychological help-seeking. Model 1 centered on females, while Model 2 focused on males. Model 3 integrated interaction terms, juxtaposing gender against each independent variable, to identify potential differential impacts on male and female cohorts. All analyses adopted a two-tailed approach. A significance level was demarcated at *P* = 0.05.

## Results

Table 1 presents the demographic profile of the participants, the mean score of GHQ-20, and the distribution of family function. Participants had an average age of 22.78 years (SD = 1.42), with females constituting 53.55%. A significant proportion, 69.04%, reported cohabiting with at least one sibling. The majority, 73.20%, recognized the usefulness of professional psychological help-seeking. In terms of their self-rated of psych knowledge, 61.84% rated themselves at a low level, while 38.16% believed they had a high level of understanding. Notably, 48.22% indicated experiencing moderately impaired or severe family dysfunction. Scores on the GHQ-20 varied between 0 and 20, with a mean of 5.16 (SD = 4.10).

**Table 1:** Characteristics of participants

Participant characteristics	*N* = 3.453	
	*n*	%
**Age (Years)** (Missing = 22)	22.78 ± 1.42	
**Gender**		
Male	1604	46.45
Female	1849	53.55
**Sibling status** (Missing = 3)		
Only-child	1068	30.96
With siblings	2382	69.04
**Major** (Missing = 4)		
Clinical medicine	519	15.05
Non-clinical medicine	2930	84.95
**Prior psycho-help experience**		
None	2863	82.91
Once	462	13.38
More than once	128	3.71
**Help-Seeking Utility Perception** (Missing = 2)		
Very useful	1267	36.71
Somewhat useful	1259	36.48
Neutral	658	19.07
Somewhat Not useful	183	5.30
Not useful at all	84	2.43
**Self-Rated Psych Knowledge** (Missing = 7)		
Low level	2131	61.84
High level	1315	38.16
**APGAR score**		
Severe family dysfunction: 0–3	252	7.30
Moderately impaired family dysfunction: 4–6	1413	40.92
Good family function: 7–10	1788	51.78
**GHQ score**	5.16 ± 4.10	

Abbreviations: APGAR, Family Care Index; GHQ, General Health Questionnaire

Table 2 delineates the attitudes towards seeking mental health help, highlighting variances between male and female respondents. On the ATSPPH-SF scale, the overall mean score was 15.04 (SD = 3.45). Notably, female participants manifested significantly higher scores than their male counterparts (15.64 vs. 14.34, *P* < 0.001). In evaluating individual statements, female students consistently exhibited more favorable attitudes, with the exceptions being items 4 and 7.

**Table 2:** Gender differences in scores on the professional psychological help-seeking attitude

Attitudes Towards Seeking Professional Psychological Help Short Form (ATSPPH-SF)	Total (*N* = 3,453)		Male (*n* = 1,604)		Female (*n* = 1,849)		P*
	*n*	%	*n*	% (95% CI)	*n*	% (95% CI)	
1. If I believed I was having a mental breakdown, my first inclination would be to get professional attention (Missing = 1)	2328	67.44	934	58.23 (55.82, 60.64)	1394	75.43 (73.47, 77.40)	**< 0.001**
2. The idea of talking about problems with a psychologist strikes me as a poor way to get rid of emotional conflicts (Missing = 2)	1400	40.57	622	38.80 (36.42, 41.19)	778	42.10 (39.85, 44.35)	**0.049**
3. If I were experiencing a serious emotional crisis at this point in my life, I would be confident that I could find relief in psychotherapy (Missing = 3)	1986	57.57	846	52.81 (50.36, 55.25)	1140	61.69 (59.47, 63.90)	**< 0.001**
4. There is something admirable in the attitude of a person who is willing to cope with his or her conflicts and fears without resorting to professional help (Missing = 5)	590	17.11	279	17.40 (15.55, 19.26)	311	16.86 (15.15, 18.56)	0.670
5. I would want to get psychological help if I were worried or upset for a long period of time (Missing = 2)	2510	72.73	1020	63.63 (61.28, 65.99)	1490	80.63 (78.83, 82.43)	**< 0.001**
6. I might want to have psychological counseling in the future (Missing = 7)	1792	52.00	741	46.31 (43.87, 48.76)	1051	56.93 (54.68, 59.19)	**< 0.001**
7. A person with an emotional problem is not likely to solve it alone; he or she is likely to solve it with professional help (Missing = 6)	1252	36.32	562	35.15 (32.81, 37.49)	690	37.34 (35.13, 39.54)	0.182
8. Considering the time and expense involved in psychotherapy, it would have to have a potential for major benefits before I would consider it worthwhile (Missing = 3)	1613	46.75	655	40.89 (38.48, 43.29)	958	51.84 (49.56, 54.12)	**< 0.0010**
9. A person should work out his or her own problems; getting psychological counseling would be a last resort (Missing = 1)	1105	32.01	429	26.76 (24.60, 28.93)	676	36.56 (34.37, 38.76)	**< 0.001**
10. Personal and emotional troubles, like many things, tend to work out by themselves	1050	30.41	393	24.50 (22.40, 26.61)	657	35.53 (33.35, 37.71)	**< 0.001**
ATSPPH-SF total score (mean ± SD)	15.04 ± 3.45		14.34 ± 3.30		15.64 ± 3.47		**< 0.001**

Note: Items 1, 3, 5, 6, and 7 are scored positively; items 2, 4, 8, 9, and 10 are reverse-scored. Percentages reflect the proportion of respondents who ‘Agree’ or ‘Strongly Agree’ with positive items and who ‘Disagree’ or ‘Strongly Disagree’ with reverse-scored items. Bold indicates a significant *p*-value of less than 0.05

Table 3 elucidates the findings from three distinct multivariate linear regression analyses. For males (Model 1), both the lower level of psych knowledge (*ß* = -0.34, *P* = 0.040) and mental health status (*ß* = -0.15, *P* < 0.001) were determinants of less favorable attitudes toward seeking help for psychological problems. Students with moderately impaired family dysfunction (*ß* = -0.71, *P* < 0.001) showed more negative attitudes towards help-seeking compared to those with good family function. Importantly, respondents perceiving some utility in seeking professional help (*ß* = 1.05, *P* < 0.001) manifested more affirmative attitudes in this context.

**Table 3:** Multivariate linear regression analysis of factors associated with attitudes towards seeking professional psychological help

Variables	Model 1: Male (*n* = 1,604)					Model 2: Female (*n* = 1,849)				Model 3: *P*-value Interaction between Gender and Variables
	Estimate	SE	t value	*P*-value		Estimate	SE	t value	*P*-value	
Constant	16.51	1.32	12.47	**< 0.001**		16.20	1.29	12.52	**< 0.001**	
**Age**	-0.07	0.06	-1.30	0.193		-0.03	0.05	-0.60	0.546	0.253
**Sibling status** (Ref = With siblings)										
Only-child	0.29	0.16	1.76	0.079		0.11	0.18	0.66	0.511	0.460
**Major** (Ref = Clinical medicine)										
Non-clinical medicine	0.09	0.22	0.42	0.677		-0.04	0.22	-0.18	0.858	0.652
**Prior psycho-help experience** (Ref = None)										
Once	-0.24	0.23	-1.02	0.306		-0.44	0.24	-1.86	0.063	0.543
More than once	0.78	0.41	1.88	0.061		1.20	0.42	2.86	**0.004**	0.474
**Help-Seeking Utility Perception** (Ref = Somewhat not useful/Not useful at all)										
Neutral	-0.45	0.31	-1.44	0.149		0.36	0.37	0.98	0.326	0.093
Somewhat useful/Very useful	1.05	0.27	3.88	**< 0.001**		1.76	0.34	5.23	**< 0.001**	0.100
**Self-Rated Psych Knowledge** (Ref = High level)										
Low level	-0.34	0.16	-2.05	**0.040**		-0.36	0.16	-2.22	**0.027**	0.914
**APGAR score** (Ref = Good family function: 7–10)										
Severe family dysfunction: 0–3	-0.51	0.31	-1.63	0.103		-0.73	0.33	-2.22	**0.027**	0.633
Moderately impaired family dysfunction: 4–6	-0.71	0.17	-4.19	**< 0.001**		-1.12	0.17	-6.63	**< 0.001**	0.080
**GHQ** score	-0.15	0.02	-7.57	**< 0.001**		-0.09	0.02	-4.56	**< 0.001**	**0.049**

Note: Bold indicates a significant *p-*value of less than 0.05

For females (Model 2), having more than once psycho-help experiences (*ß* = 1.20, *P* = 0.004) and a favorable perception of the utility of professional help-seeking (*ß* = 1.76, *P* < 0.001) were positively correlated with attitude towards seeking professional help. In contrast, worse family function (severe family dysfunction: *ß* = -0.73, *P* = 0.027; moderately impaired: *ß* = -1.12, *P* < 0.0001), poorer psych knowledge (*ß* = -0.36, *P* = 0.027), and lower level of mental health (*ß* = -0.09, *P* < 0.001) were significantly associated with more negative attitude towards seeking help. In Model 3, a notable interaction emerged between gender and the GHQ score (*P* = 0.049).

## Discussion

In our study, the mean score of ATSPPH-SF scale was 15.04 ± 3.45, indicating that the attitude towards seeking professional psychological help was relatively negative among Chinese medical students. Compared with the results in Chinese community-dwelling population (18.13 ± 5.63) [38], Chinese medical students had even more negative attitudes towards seeking psychological help. In addition, the total score on ATSPPH-SF scale in this survey was also lower than some European countries, such as Germany (17.2 ± 4.4), Ireland (18.4 ± 5.4) and Portugal (20.0 ± 5.7) [39], which indicated more negative attitudes toward psychological help-seeking among Chinese medical students. Given the gaps, it is essential to explore the factors influencing help-seeking attitudes.

In parallel with the literature, our research found that women showed more positive attitudes toward seeking professional psychological help than men [40]. Specifically, statistically significant gender differences were found in 8 items, all of which showed that women had a more positive attitude. This gender gap may be explained as a consequence of traditional gender norms. It is widely recognized that women are more emotional than men. Women tend to be more willing to disclose their emotions, and the association between distress disclosure and positive help-seeking attitudes had been well established [41]. Besides, as early as 1981, Kessler et al. had noted that women were more likely to seek psychiatric help than men, and attributed this difference to the fact that that women were more inclined than men to translate nonspecific feelings of psychiatric distress into conscious recognition [42]. However, influenced by the prevailing masculinity in the social environment, men’s attitudes towards seeking help were more negative [43]. Hegemonic masculinity is characterized by emotional control and bravery [44], while suffering from psychological distress, such as depression, is usually considered powerless and uncompetitive. Therefore, in order to demonstrate and strengthen masculinity, men tend to hold a rejection attitude towards emotional disclosure, let alone seek professional help.

The multivariable linear regression models suggested that the Family APGAR Index was a predictor of ATSPPH-SF for both male and female groups, indicating that students with lower family care are more likely to hold a negative attitude towards professional psychological help-seeking. This is consistent with previous research among Chinese community population [38]. As an important form of social support, family care plays an irreplaceable role in Chinese students’ decision-making. A survey has shown that when people experienced depression symptoms, family members were the first choice for most people to seek help from, and most importantly, mothers would provide advice on problem-solving [45]. Considering this, we can believe that family members have a direct impact on people’s attitudes towards seeking professional psychological help.

Utilizing the GHQ-20 for assessment, our study discerned that higher cumulative scores among Chinese students—both male and female—indicative of increased psychological distress, were associated with progressively negative attitudes towards seeking professional help. This observation raises concerns, especially given that those most in need of psychological interventions appear more hesitant to pursue them. Past research has pinpointed an association between elevated psychological distress and heightened stigma scores [46]. Numerous studies consistently highlight a marked inverse relationship between stigma and the propensity to seek professional psychological assistance [38, 47]. Furthermore, an evaluation involving 220 medical students revealed a significant indirect influence of mental distress on help-seeking attitudes, mediated by self-stigma [48]. These findings are in alignment with our own.

Our findings are congruent with extant literature, underscoring a pronounced negative association between a diminished self-assessment of psych knowledge and attitudes towards procuring mental health support. A limited self-assessment of psychological health knowledge often implies a lack of confidence in recognizing mental disorders’ symptoms, potential misconceptions or biases about psychological health, and possibly a reluctance or skepticism towards professional therapeutic interventions. Empirical evidence from the Western Pacific region substantiates this connection, indicating that those with a more informed self-perception of depression knowledge exhibit a more positive orientation toward mental health interventions [49]. This pattern echoes prior findings in the Chinese context [38, 50]. Importantly, our analysis reveals that students acknowledging the merits of psychological interventions, such as counseling and pharmacotherapy, manifest a more open-minded perspective on their utility. This underscores the critical role of rectifying misconceptions and enhancing accurate self-evaluation of one’s psychological health literacy to foster more constructive help-seeking behaviors.

Our study elucidated a gender-specific discrepancy in the correlation between prior psycho-help experiences and attitudes towards seeking psychological assistance. Among female university students, a pronounced positive association was identified: those with prior psycho-help experiences displayed a more favorable disposition towards future psychological interventions. This suggests that initial therapeutic encounters potentially frame subsequent perceptions and receptivity in females [22, 51]. Conversely, such a relationship was absent among their male counterparts. This could be attributed to entrenched socio-cultural norms, which often encourage males to mask their emotional vulnerabilities, thereby rendering them less susceptible to the influences of past therapeutic interactions [48]. These findings accentuate the paramount importance of gender considerations in mental health practices and underscore the necessity for tailored approaches when addressing the mental health needs of distinct gender groups. Recognizing these gender differences, we suggest targeted interventions to support male and female medical students differently. For females, amplifying existing positive attitudes through educational programs and peer support can be beneficial. For males, addressing societal stigmas around emotional vulnerability and promoting models of healthy masculinity that include seeking help could mitigate negative perceptions. Tailored strategies that consider these gender-specific nuances are crucial for improving psychological help-seeking attitudes among all students.

Our study is underscored by several limitations that warrant acknowledgment. To begin with, the cross-sectional nature of our research design precludes the determination of definitive causal relationships between attitudes towards psychological help-seeking and their potential determinants. This inherent limitation accentuates the need for future longitudinal investigations to ascertain causality and corroborate our preliminary findings. Additionally, our study’s sample is predominantly sourced from a single province in the southernmost region of China. While this provides a nuanced understanding of the specific demographic, it simultaneously raises concerns about the generalizability of our results. It is prudent to approach our findings with caution when extrapolating to broader populations or diverse cultural contexts. Future research endeavors spanning a more diverse geographic scope would provide a more comprehensive insight into this domain.

## Conclusion

This study is pioneering in examining gender disparities in attitudes toward seeking professional psychological help and associated factors among Chinese medical students. Notably, our findings reveal a significant gender difference, with male students exhibiting markedly more negative attitudes compared to their female counterparts. Moreover, psych knowledge, mental health status, family function, help-seeking utility perception and psycho-help experiences were identified as factors influencing attitudes toward seeking help. Given this gender difference, there is an urgent need for targeted intervention measures and support systems to address the challenges male medical students face in accessing mental health services.

## Data Availability

The datasets used in this study will be made available when requested to the corresponding author.
